# Type I Interferon Regulates the Survival and Functionality of B Cells in Rainbow Trout

**DOI:** 10.3389/fimmu.2020.01494

**Published:** 2020-07-09

**Authors:** Ottavia Benedicenti, Tiehui Wang, Esther Morel, Christopher J. Secombes, Irene Soleto, Patricia Díaz-Rosales, Carolina Tafalla

**Affiliations:** ^1^Animal Health Research Center (CISA-INIA), Madrid, Spain; ^2^Scottish Fish Immunology Research Centre, School of Biological Sciences, University of Aberdeen, Aberdeen, United Kingdom

**Keywords:** teleost fish, B cells, interferon (IFN), IgM, phagocytosis

## Abstract

Interferons (IFNs) orchestrate antiviral responses in jawed vertebrates and can be classified into three types based on different aspects of their genomic organization, structure and receptors through which they signal and function. Generally, type I and type III IFNs include cytokines that directly induce an antiviral response, whereas type II IFNs are well-known for their immunomodulatory role during viral infections. In mammals, type I IFNs have been shown to also regulate many aspects of B cell development and differentiation. Yet, these functions have been only faintly investigated for teleost IFNs. Thus, in the current study, we have examined the effects of a model type I rainbow trout IFN molecule (IFNa) on blood naïve (IgM^+^IgD^+^) B cells, comparing them to those exerted by type II IFN (IFNγ). Our results demonstrate that IFNa increases the survival of naïve rainbow trout B cells, in the absence of lymphoproliferative effects, by rescuing them from spontaneous apoptosis. Additionally, IFNa increased the phagocytic capacity of blood IgM^+^IgD^+^ B cells and augmented the number of IgM-secreting cells in blood leukocyte cultures. IFNγ, on the other hand, had only minor effects up-regulating IgM secretion, whereas it increased the phagocytic capacity of IgM^−^ cells in the cultures. Finally, given the recent identification of 9 *mx* genes in rainbow trout, we have also established which of these genes were transcriptionally regulated in blood naïve B cells in response to IFNa. This study points to a previously undescribed role for teleost type I IFNs in the regulation of B cell responses.

## Introduction

Interferons (IFNs) are potent antiviral cytokines induced during the course of viral infections categorized into three classes (types I, II and III) on the basis of the receptors used, their genomic organization, sequence or structural homology (1). Although type I and type II IFNs were originally distinguished on the basis of the cells responsible for their production, it is now evident that they differ at many levels. Both types of cytokines are implicated in the response to viral infection, but while type I IFNs are well-known for their antiviral effects, type II IFNs play immunoregulatory functions during infection (2, 3). Thus, type I IFNs comprise a large family of closely related cytokines classified in mammals as IFNα, IFNβ, IFNε, IFNκ, IFNω, IFNδ, IFNζ, and IFNτ (3, 4). These cytokines are produced by almost every cell type upon the recognition of a virus or a virus -like stimulus. Once secreted, these IFNs bind to their cell surface receptor and initiate a signaling cascade that eventually leads to the transcriptional regulation of hundreds of IFN-stimulated genes (ISGs) [reviewed in (5)]. Among these type I IFN-induced proteins, many of them such as Myxovirus resistance (Mx) proteins directly interfere with viral replication (6). On the other hand, IFNγ, the only type II IFN found in mammals, is secreted almost exclusively by NK and T lymphocytes in response to certain cytokines (7). IFNγ has been shown to induce nitric oxide (NO) production, phagocytosis, and secretion of pro-inflammatory cytokines of macrophages, promoting their polarization to an M1 phenotype (8). IFNγ also plays an important role in antigen presentation, as it increases MHC II expression and maturation of macrophages and dendritic cells (DCs) (8, 9). Interestingly, although only a limited subset of cells express IFNγ, its receptor is ubiquitously expressed, and through this interaction, IFNγ is also capable of inducing antiviral effects similar to those elicited by type I IFNs (10). Mammalian type III IFNs, on the other hand, constitute a group of four cytokines that activate a signaling pathway similar to that of type I IFNs through a specific receptor (11, 12).

In addition to their direct antiviral effects, it has become evident in mammals that type I IFNs also play pleiotropic effects during the development and differentiation of B cells (13). In mice, for example, IFNα/β has been shown to increase the survival of B cells through a decrease of Fas-mediated apoptosis (14, 15), and to enhance responses to B cell receptor (BCR) ligation such as calcium mobilization, IgM internalization, induction of activation markers or proliferation (14). Additionally, type I IFNs significantly augment the response of B cells to Toll-like receptor 7 (TLR7) ligands (16), and synergize with interleukin 6 (IL6) to promote the differentiation of B cells to antibody-secreting plasma cells (17). Thus, mammalian type I IFNs impact B cell functions through a wide range of direct effects or indirectly by regulating DCs or macrophages that in turn affect B cell functionality (13, 18, 19). In this context, it is not surprising that the progression of systemic autoimmune diseases such as systemic lupus erythematosus (SLE) often associates with an increased type I IFN production (13), as IFN levels in SLE patients correlate with autoantibody production (20).

Teleost fish also possess multiple type I IFNs, varying in number depending on the fish species [reviewed in (21)]. The number of type I IFN genes is especially high in particular teleost lineages such as salmonids, potentially as a consequence of the additional whole genome duplication (WGD) event that took place throughout evolution in this lineage. Fish type I IFNs can be phylogenetically divided into three major groups (22), classified into 6 subgroups (a, b, c, d, e, and f) in salmonids. IFNa, IFNd, and IFNe are in Group I, IFNb and IFNc are in Group II, whilst IFNf is in Group III, with multiple genes present for most subgroups (23). It is important to clarify that IFNa and IFNb are not orthologs of mammalian IFNα and IFNβ. To date, the antiviral activity of several teleost type I IFNs has been confirmed, including IFNa, IFNb, and IFNc (24–26). In contrast, IFNd showed little or no antiviral activity in salmon (or zebrafish) (25, 26). Whilst there is a single gene for IFNγ in most vertebrate groups, again as a consequence of WGD, two genes are found in salmonids and cyprinids (27, 28). Although few functional studies have been undertaken in these species as to whether differences exist between the two IFNγ paralogs, several independent transcriptional studies show no major differences concerning their regulatory mechanisms (27, 29–31). Interestingly, some teleost species [reviewed in (32)] have an additional IFNγ-like gene designated as IFNγ-rel that presumably originated from a teleost-specific tandem duplication. With regard to IFNγ and IFNγ-rel bioactivity, most assays conducted thus far in teleosts have been predominantly focused on analyzing its effects on macrophages [reviewed in (32)].

In teleost fish, three Ig classes have been described to date, IgM, IgD, and IgT (a teleost-specific Ig) which define different B cell subsets. IgM^+^IgD^+^ constitute the main B cell type in central immune tissues such as peripheral blood, spleen and head kidney (the main hematopoietic organ). As described in mammals, upon activation, these naïve cells loose surface IgD expression becoming IgM^+^IgD^−^ cells (33). Additionally, as also reported in specific human and mice mucosal compartments (34–36), B cells exclusively expressing IgD on the cell surface have been identified in catfish (*Ictalurus punctatus*) blood (37) and rainbow trout (*Oncorhynchus mykiss*) gills (38) and intestine (39). The function of these cells is still unknown. IgT^+^ cells, on the other hand, constitute a distinct B cell lineage that seems to be specialized in mucosal responses, as the ratio of IgT^+^ B cells to IgM^+^IgD^+^ B cells is higher in mucosal surfaces, and IgT responses have been shown to be restricted to mucosal compartments in response to some infections (40, 41). Interestingly, fish IgM^+^IgD^+^ B cells have been shown to share many phenotypic and functional characteristics of mammalian B1 cells (42, 43), innate B cells that immediately respond to antigens to produce natural antibodies that interfere with pathogen replication until a specific immune response is mounted (44). In this context, it seemed quite relevant to establish how fish naïve IgM^+^IgD^+^ B cells respond to innate signals produced during the early stages of a pathogenic exposure such as IFNs.

To date, whether type I or type II IFNs affect B cell functionality has been only addressed in fish in one study in which flounder (*Paralichthys olivaceus*) type I IFN was shown to enhance the phagocytic capacity of IgM^+^ B cells as well as their production of reactive oxygen species (ROS) (45). In the current study, we have investigated the effects of both type I and type II IFN on IgM^+^ B cells using rainbow trout (*Oncorhynchus mykiss*) as a model species. For this, we have stimulated rainbow trout peripheral blood leukocytes (PBLs) with recombinant trout IFNa and IFNγ (rIFNa and rIFNγ) and then studied different functions of naïve B cells. We chose to undertake these studies with PBLs, as in rainbow trout, the blood is where the higher percentage of IgM^+^IgD^+^ B cells are found, ~44% (46). Our results demonstrate that while IFNa regulates many aspects of B cell functionality, type II IFN only has minor effects on IgM secretion. The effects of trout IFNa on naïve B cells include increased survival through decreased apoptosis, up-regulated phagocytic capacity and augmented IgM secretion and Mx transcription. However, in contrast to mammalian type I IFN, trout IFNa was not capable of synergizing with BCR signaling. This study thus demonstrates that, in addition to their direct antiviral effects, teleost type I IFNs play an important role in the regulation of B cell responses as in mammals. The differences between the effects exerted in mammals and fish will most probably be a consequence of the significant differences that exist between mammalian conventional B cells and fish B cells.

## Materials and Methods

### Experimental Fish

Healthy rainbow trout (*Oncorhynchus mykiss*) of ~50–70 g were obtained from Piscifactoria Cifuentes (Cifuentes, Guadalajara, Spain) and maintained at the animal facilities of the Animal Health Research Center (CISA-INIA, Spain) in an aerated recirculating water system at 15°C, with a 12:12 h light: dark photoperiod. Fish were fed twice a day with a commercial diet (Skretting, Spain). Prior to any experimental procedure, fish were acclimatized to laboratory conditions for at least 2 weeks. During this period no clinical signs of disease were ever observed.

### Leukocyte Isolation

Rainbow trout were killed with benzocaine (Sigma-Aldrich) and blood was extracted with a heparinized needle from the caudal vein. Blood was diluted 10 times with Leibovitz's medium (L-15, Gibco) containing 100 I.U./ml penicillin and 100 μg/ml streptomycin (P/S, Life Technologies), 10 I.U./ml heparin and 5% fetal calf serum (FCS, Thermo Fisher Scientific) and then placed onto 51% Percoll (GE Healthcare) density gradients. Cell suspensions were centrifuged at 400 × *g* for 30 min at 4°C, the interface cells were collected and washed with L-15 supplemented with antibiotics and 5% FCS. The viable cell concentration was determined by Trypan blue (Sigma-Aldrich) exclusion and cells were resuspended in L-15 with 5% FCS at a concentration of 2 × 10^6^ cells/ml.

### Production of Recombinant IFNs

rIFNa and rIFNγ were produced as described previously (47, 48). Both recombinant proteins were expressed in *Escherichia coli* BL21 Star (DE3) by isopropyl β-D-1-thiogalactopyranoside (IPTG) induction and purified under denaturing conditions with extensive washing with buffer containing Triton X-100 to remove lipopolysaccharide (LPS) as described previously. The purified proteins were refolded in a buffer containing 0.5 M arginine, and re-purified under native conditions (47–49). The bioactivity was established by testing their ability to induce the expression of specific target genes, such as Mx and CXCL11_L1 *in vitro* in rainbow trout cell lines such as the monocyte/macrophage rainbow trout cell line RTS11 (47, 48). Both proteins had no effects on the expression of known LPS-responsive genes, such as IL1β and cathelicidin-1 in RTS11 cells (50), confirming the lack of LPS contamination.

### Cell Stimulation

Peripheral blood leukocytes (PBLs), suspended in L-15 medium supplemented with antibiotics and 5% FCS, were dispensed into 24 (2 × 10^6^ cells/well) or 96-well plates (4 × 10^5^ cells/well) (Nunc), depending on the experiment. The rIFNa and rIFNγ were used at a final concentration of 50 and 20 ng/ml, respectively, after establishing that these were the concentrations that rendered maximal effects in terms of B cell survival and *mx* gene expression (data not shown). These concentrations are in accordance with previous results (47, 48, 51). Controls incubated with media alone were included in all experiments. Leukocytes were cultured at 20°C for different times, depending on the experiment.

### Flow Cytometry

Cells were stained with anti-trout IgM [1.14 mAb mouse IgG1 coupled to R-phycoerythrin (R-PE), 0.25 μg/ml], anti-trout IgD [mAb mouse IgG1 coupled to allophycocyanin (APC), 4 μg/ml] and anti-trout MHC II β-chain [mAb mouse IgG1 coupled to fluorescein isothiocyanate (FITC), 4 μg/ml] for 1 h at 4°C, as previously described (52–54). Antibodies were fluorescently labeled using R-PE, APC or FITC Lightning-Link labeling kits (Innova Biosciences) following the manufacturer's instructions. After the staining, cells were washed twice with staining buffer (phenol red-free L-15 medium supplemented with 2% FCS). The cell viability was checked by addition of 4',6-diamine-2'-phenylindole dihydrochlorid (DAPI, 0.2 μg/ml). Cells were analyzed on a FACS Celesta flow cytometer (BD Biosciences) equipped with BD FACSDiva™ software. Flow cytometry analysis was performed with FlowJo V10 (TreeStar).

### Leukocyte Proliferation

The Click-iT™ Plus EdU Alexa Fluor™ 488 Flow Cytometry Assay Kit (Invitrogen™) was used to measure the proliferation of IgM^+^IgD^+^ B cells following the manufacturer's instructions. PBLs were incubated for 3 days at 20°C in 96-well plates with the rIFNs or media alone. In some experiments, PBLs were also stimulated with unlabelled monoclonal antibody (mAb) against trout IgM (clone 1.14, mouse IgG1) at a final concentration of 10 μg/ml, to induce cross-linking of the BCR as described previously (43). After 3 days, 0.1 μM of 5-ethynyl-2'-deoxyuridine (EdU) was added to the cultures that were further incubated for 24 h. Thereafter, cells were collected and stained with the LIVE/DEAD® Fixable Dead Cell Stain Kit (Invitrogen™) for 30 min at 4°C (protected from light) to check cell viability following the manufacturer's instructions. Subsequently the cells were stained with anti-trout IgM (1.14 mAb mouse IgG1 coupled to R-PE, 0.25 μg/ml) and anti-trout IgD (mAb mouse IgG1 coupled to APC, 4 μg/ml) for 1 h at 4°C, as described above, and analyzed on a FACS Celesta flow cytometer.

### Apoptosis

The apoptosis assay was performed using the PE Annexin V Apoptosis Detection Kit I (BD Pharmingen™) following the manufacturer's instructions. Briefly, PBLs were incubated for 48 or 72 h at 20°C in 96-well plates with the rIFNs or with media alone. Thereafter, cells were stained with anti-trout IgM [1.14 mAb mouse IgG1 coupled to FITC, 0.425 μg/ml] for 20 min at 4°C. After washing with staining buffer, the cells were centrifuged at 400 × *g* for 5 min at 4°C, and 1.5 μl of PE Annexin V and 4 μl of 7-aminoactinomycin D (7-AAD) per 200 μl of 1X Annex V Binding Buffer added to the cells. The cells were then incubated for 15 min at room temperature (RT) in the dark before being transferred to flow cytometry tubes (Falcon® 5 mL Round Bottom Polystyrene Test Tube, Corning) containing 400 μl of 1X Annex V Binding Buffer, and analyzed within 1 h on a FACS Celesta flow cytometer.

### Phagocytic Activity

PBLs were seeded into 24-well plates and incubated for 72 h at 20°C with the rIFNs or media alone. The cells were then collected and resuspended in L-15 medium supplemented with antibiotics without serum and incubated for 3 h at 20°C with fluorescent beads (FluoSpheres™ Carboxylate-Modified Microspheres, 1.0 μm, crimson fluorescent (625/645), 2% solids, Invitrogen™) at a cell: bead ratio of 1:10, as described before (55). After the incubation period, cells were harvested by gently pipetting, and non-ingested beads were removed by centrifugation (100 × *g* for 10 min at 4°C) over a cushion of 3% (weight/volume) bovine serum albumin (BSA) lyophilized powder, ≥96% (Sigma-Aldrich) in PBS supplemented with 4.5% (weight/volume) D(+)-glucose monohydrate (Merck). Cells were then resuspended in staining buffer, labeled with anti-IgM-FITC (1.14) (0.425 μg/ml) and anti-IgD-PE (40 μg/ml) and analyzed on a FACS Celesta flow cytometer. The cell viability was checked by staining the cells with DAPI (0.2 μg/ml). In some experiments, cytochalasin B (0.05 μg/ml) was added to the cells immediately before the addition of the beads to verify active phagocytosis as described previously (55). Flow cytometry analysis was performed with FlowJo V10 to calculate the ratio between the percentage of phagocytic IgM^+^ B cells among the total IgM^+^ B cell population, the ratio between the percentage of highly phagocytic IgM^+^ B cells among the total IgM^+^ phagocytic B cell population and the mean fluorescence intensity (MFI) of internalized beads in all IgM^+^ phagocytic cells.

### ELISPOT Assay

PBLs from individual fish were stimulated with rIFNs for 72 h at 20°C or left unstimulated in the same conditions in 96-well plates. ELISPOT plates containing Inmobilon-P membranes (Millipore) were activated with 70% ethanol for 30 s, coated with an anti-IgM mAb (clone 4C10) at 2 μg/ml in phosphate buffer saline (PBS) and incubated overnight at 4^?^C. To block non-specific binding to the membrane, plates was incubated with 2% BSA in PBS for 2 h at RT. At this point, cells that had been stimulated with rIFNs for 72 h or unstimulated cells were transferred to the ELISPOT plates (5 × 10^4^ cells/well) and left overnight at 20°C. The day after, cells were washed away five times with PBS and plates blocked again with 2% BSA in PBS for 1 h at RT. After blocking, biotinylated anti-IgM mAb (clone 4C10) was added to the plates and incubated at 1 μg/ml for 1 h at RT. Following five washing steps (in PBS), the plates were developed using streptavidin-HRP (Thermo Scientific) for 1 h at RT, washed again with PBS and incubated with 3-amino-9-ethylcarbazole (Sigma-Aldrich) for 30 min at RT in the dark. Substrate reaction was stopped by washing the plates with tap water. Once the membranes had dried, they were digitally scanned and the number of spots in each well-determined using an AID iSpot Reader System (Autoimmun Diagnostika GMBH).

### Cell Sorting

PBLs were seeded into 24-well plates and incubated for 24 h with rIFNs or with media alone at 20°C. At this point, cells were collected and incubated for 1 h at 4°C with anti-IgM-FITC and anti-trout IgD-APC as described above. Following several washing steps, cells were resuspended in staining buffer and IgM^+^IgD^+^ B cells isolated by flow cytometry using a BD FACSAria III cell sorter (BD Biosciences) based on their FSC/SSC profile and then on the basis of the fluorescence emitted by the anti-IgM and anti-IgD antibodies. Approximately 70,000 IgM^+^IgD^+^ B cells were collected in PBS for subsequent RNA isolation.

To confirm a direct effect of rIFNs on IgM^+^IgD^+^ B cell survival, PBLs were incubated for 1 h at 4°C with a biotinyilated Fab fragment of anti-IgM 1.14 (to avoid cell activation) in staining buffer. Following two washing steps, Streptavidin-Phycoerythrin (PE) (BD Pharmingen) was added. After 20 min at 4°C, cells were resuspended in staining buffer and IgM^+^IgD^+^ B cells isolated as described above. Sorted IgM^+^IgD^+^ B cells were then incubated with rIFNs or media alone for 3 days at 20°C. After this time, cells were counterstained with 0.2 μg/ml DAPI, and analyzed on a FACS Celesta flow cytometer.

### Real Time PCR Analysis

Total RNA was isolated from FACS sorted IgM^+^IgD^+^ B cells using the Power SYBR Green Cells-to-Ct Kit (Invitrogen), following the manufacturer's instructions. Briefly, total RNA was treated with DNase during the process to remove genomic DNA that might interfere with the PCR reactions and then reverse transcribed into cDNA following the manufacturer's instructions. To evaluate the levels of transcription of the different genes, real time RT-PCR was performed with a LightCycler® 96 System instrument (Roche) using SYBR Green PCR core Reagents (Applied Biosystems) and specific primers (Table S1). Samples obtained from individual fish were analyzed in duplicate under the following conditions: 1 min at 95°C, followed by 45 amplification cycles (15 s at 95°C and 1 min at 60°C). A melting curve for each primer set was obtained by reading fluorescence every degree between 60 and 95°C to ensure that only a single PCR product had been amplified. The expression of individual genes was normalized to the relative expression of the housekeeping gene β-actin, and the expression levels were calculated using the 2^(^−ΔCt) method, where ΔCt is determined by subtracting the β-actin value from the target Ct (Ct cut-off set to 38). This housekeeping gene was selected after verifying that no statistical differences were detected among β-actin Ct values obtained from different samples. Negative controls with no template and *minus* reverse transcriptase controls were included in all the assays.

### Statistical Analysis

Data handling, analysis and graphic representation were performed using GraphPad Prism version 7.00 for Windows, GraphPad Software, La Jolla California USA (www.graphpad.com). Statistical analyses were performed using a two-tailed Student's *t-*test and the differences between the mean values were considered significant when *P* ≤ 0.05.

## Results

### Type I IFNa Increases the Survival of IgM^+^IgD^+^ B Cells by Decreasing Their Spontaneous Apoptosis

**Figure 1:**
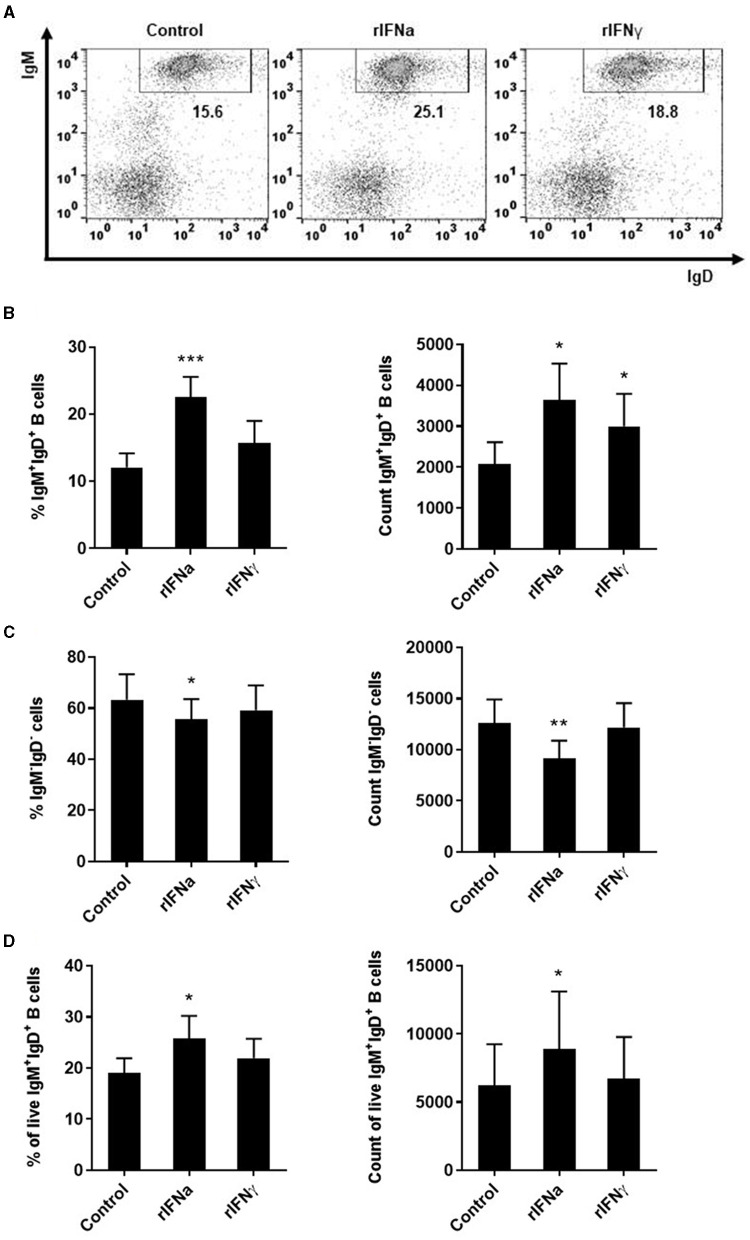
Survival of blood IgM^+^IgD^+^ B cells in response to type I and type II IFNs. PBLs were stimulated with 50 ng/ml rIFNa, 20 ng/ml rIFNγ or media alone (control) and cultured at 20°C for 72 h. Leukocytes were then labeled with specific monoclonal antibodies against trout IgM and IgD and analyzed by flow cytometry. Cells were gated on the basis of their FSC and SSC and percentages of IgM^+^IgD^+^ cells determined on singlet and live (DAPI negative) cells. Representative dot plots from one individual fish are shown **(A)** along with mean percentages and total number of cells detected for IgM^+^IgD^+^ B cells **(B)** and IgM^−^IgD^−^ cells **(C)** (mean + SEM; *n* = 9). In an independent experiment, B cells were sorted from blood leukocytes using a biotinyilated Fab fragment of anti-IgM 1.14 and then incubated with the rIFNs as described above. After 72 h, the percentage of live IgM^+^IgD^+^ B cells and the total number of live IgM^+^IgD^+^ B cells determined by flow cytometry as described in the Materials and Methods section (mean + SEM; *n* = 7) **(D)**. Asterisks denote significant differences between samples treated with rIFNs and control samples (*P ≤ 0.05, **P ≤ 0.01, ***P ≤ 0.001).

Our results show that the incubation of rainbow trout PBLs with rIFNa significantly increased the survival of IgM^+^IgD^+^ B cells after 72 h (Figures 1A,B). In this and all other flow cytometry experiments described throughout the paper with the exception of the apoptosis assays, doublets and dead cells were excluded from the analysis following the gating strategy described in Figure S1. rIFNa significantly increased both the percentage of IgM^+^ B cells and the absolute number of IgM^+^ B cells in the cultures (Figures 1A,B), while rIFNγ only had a significant effect increasing the absolute number of IgM^+^ B cells in the cultures but had no effect on the percentage of IgM^+^ B cells (Figures 1A,B). rIFNγ had no effect on the survival of the IgM^−^ leukocyte population, whereas rIFNa provoked a slight but significant down-regulation of the percentage and the total number of IgM^−^ cells in the cultures (Figures 1A,C). In these experiments, rIFNa and rIFNγ were used at their optimal concentrations (50 ng/ml and 20 ng/ml, respectively), as established before (47, 48, 51), and in preliminary experiments, where in the case of rIFNγ higher concentrations (50 ng/ml) had similar or even lower effects on IgM^+^IgD^+^ B cells (Figure S2). As this effect of rIFNa on IgM^+^IgD^+^ B cells could have been indirect, we repeated the experiments in such a way that IgM^+^IgD^+^ B cells were first sorted with an anti-IgM Fab and then stimulated with the rIFNs. The purity of the sorted B cell population was evaluated by flow cytometry (Figure S3). In this case again, only rIFNa significantly increased the survival of IgM^+^IgD^+^ B cells, demonstrating a direct effect of this cytokine on survival (Figure 1D).

**Figure 2:**
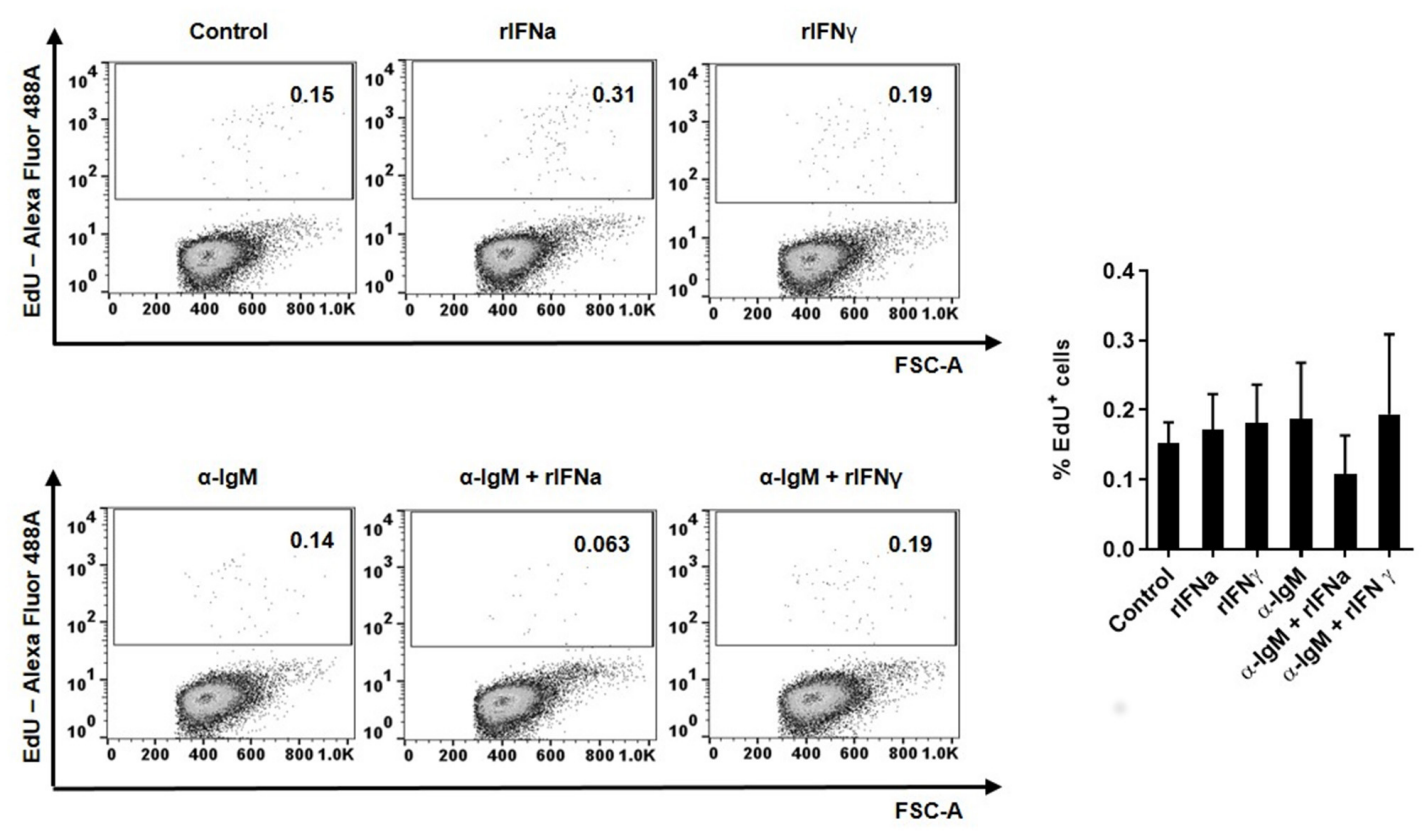
Proliferative effects of type I and type II IFNs on blood IgM^+^IgD^+^ B cells. The lymphoproliferative effects of rIFNa and rIFNγ were determined by incubating PBLs at 20°C with 50 ng/ml rIFNa, 20 ng/ml rIFNγ or media alone. For each condition, wells containing 10 μg/ml of unlabelled anti-IgM were also included. After 72 h, cells were labeled with EdU (1 μM) and incubated for a further 24 h. At that point, cells were labeled with anti-IgM and anti-IgD mAbs and the percentage of IgM^+^IgD^+^ B cells with incorporated EdU (proliferating cells) determined as described in the Materials and Methods section. Representative dot plots are presented along with a graph showing the quantification of proliferating IgM^+^IgD^+^ B cells (mean + SEM; *n* = 8).

To determine whether this increased survival of B cells in the presence of rIFNa was a consequence of lymphoproliferative effects of this cytokine, we analyzed the proliferative effect of rIFNa and rIFNγ on their own or when combined with BCR cross-linking (through incubation with anti-IgM). Our results clearly showed that none of the rIFNs exerted significant proliferative effects on rainbow trout blood IgM^+^IgD^+^ B cells by themselves (Figure 2). No synergistic effects were observed between BCR cross-linking and rIFN stimulation (Figure 2).

**Figure 3:**
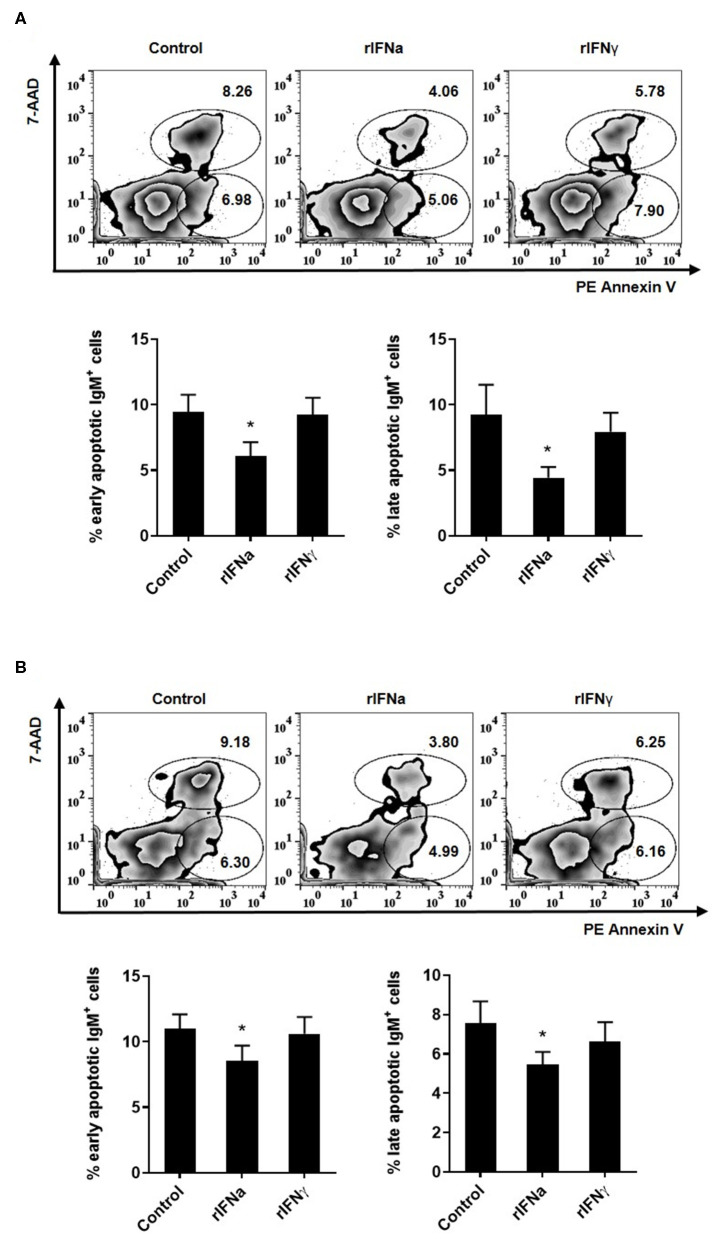
Effect of type I and type II IFNs on the spontaneous apoptosis of blood IgM^+^IgD^+^ B cells. PBLs were incubated for 48 h **(A)** or 72 h **(B)** at 20°C with 50 ng/ml rIFNa, 20 ng/ml rIFNγ or media alone (control). Then, cells were stained with anti-trout IgM for 20 min at 4°C. After washing, PE Annexin V and 7-AAD were used to stain the cells and identify early (Annexin V^+^/7-AAD^−^) and late (Annexin V^+^/7-AAD^+^) apoptosis. Data were analyzed within 1 h as described in the Materials and Methods section. Representative dot plots are presented along with graphs showing the quantification of apoptotic IgM^+^ B cells (mean + SEM; *n* = 6–9). Asterisks denote significant differences between samples treated with rIFNs and control samples (*P ≤ 0.05).

Another possible mechanism through which rIFNa could increase the survival of IgM^+^IgD^+^ B cells may be through the modulation of spontaneous apoptosis of these cells. To test this, we studied apoptosis of IgM^+^IgD^+^ B cells after 48 or 72 h of incubation with the different rIFNs. In these experiments, the leukocyte population was gated and doublets excluded from the analysis as described in Figure S1, although in this case the viability marker used was 7-AAD. Both after 48 and 72 h, a significant decrease in the percentage of early (Annexin V^+^/7-AAD^−^) and late (Annexin V^+^/7-AAD^+^) apoptotic IgM^+^IgD^+^ B cells was observed after stimulation with rIFNa (Figures 3A,B). Interestingly, the percentages of early and late apoptotic IgM^+^IgD^+^ B cells were quite similar after 48 and 72 h suggesting that B cells are mostly killed during the first 48 h of culture, whereas those that survive after 48 h remain viable for longer time periods. This effect was not observed in response to rIFNγ. Altogether, our results demonstrate that rIFNa increases the survival of blood naïve B cells, rescuing them from spontaneous apoptosis.

### Type I IFNa Increases the Phagocytic Activity of IgM^+^IgD^+^ B Cells

**Figure 4:**
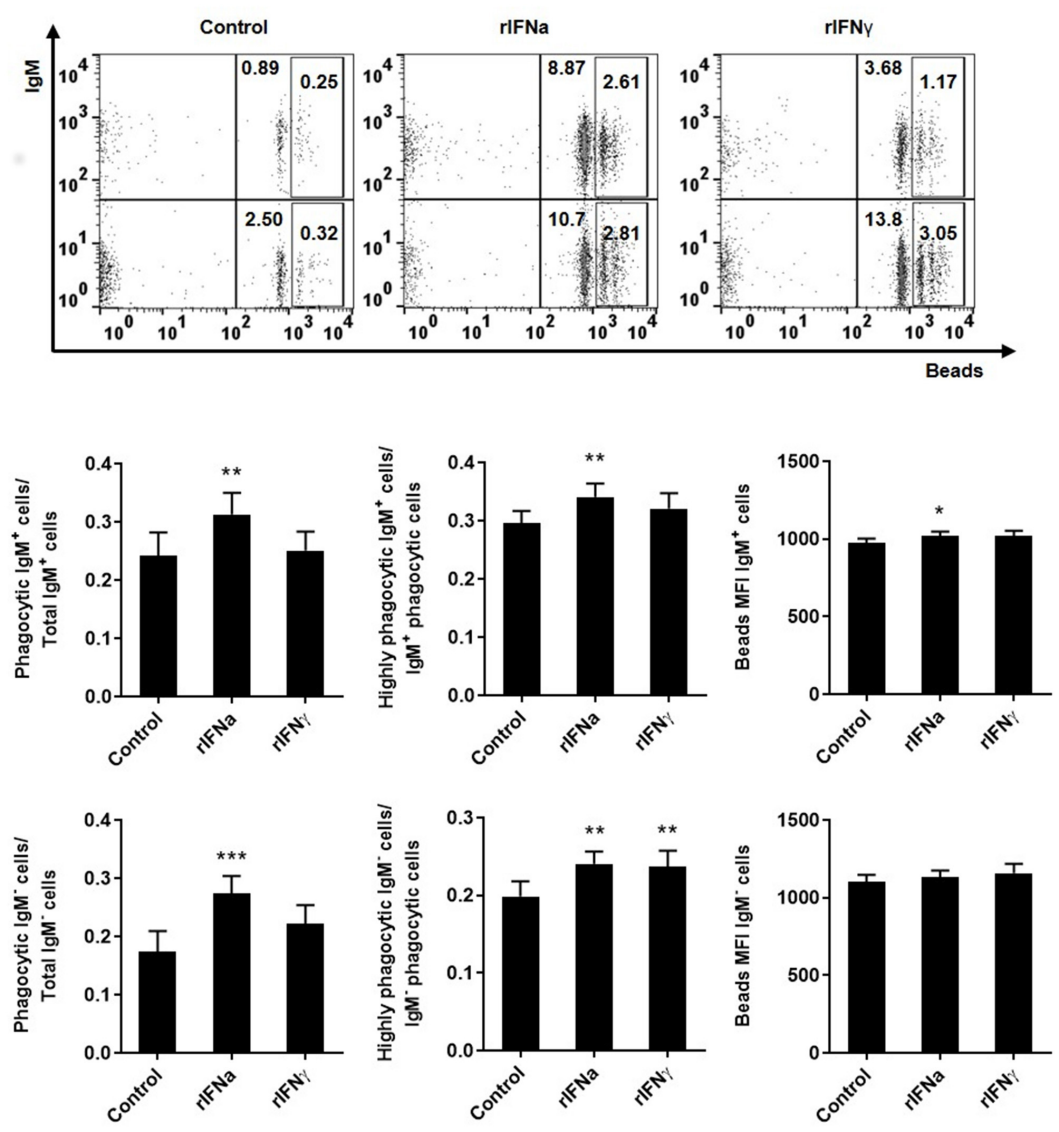
Effect of type I and type II IFNs on the phagocytic capacity of blood IgM^+^IgD^+^ B cells. PBLs were incubated for 72 h at 20°C with 50 ng/ml rIFNa, 20 ng/ml rIFNγ or media alone (control). After this time, cells were incubated with crimson red fluorescent beads (1 μm diameter) at a ratio of 1:10 (cells/ beads) for a further 3 h at 20°C and the phagocytic capacity of B cells established as described in materials and methods. Representative dot plots for each experimental condition are shown. The rectangular areas contain the highly phagocytic cell subpopulations (cells that have internalized a higher number of beads) among IgM^+^ (upper) or IgM^−^ (lower) cell sub-populations. Upper graphs show the percentage of phagocytic IgM^+^ B cells within the total IgM^+^ B cell population (left), the percentage of highly IgM^+^ phagocytic cells within the total phagocytic IgM^+^ B cell population (middle) and the mean fluorescence intensity (MFI) of internalized beads within phagocytic IgM^+^ B cells (right). Lower graphs show the same information for the IgM^−^ population. Data are means + SEM; *n* = 10. Asterisks denote significant differences between samples treated with rIFNs and control samples (*P ≤ 0.05, **P ≤ 0.01, ***P ≤ 0.001).

As teleost B cells have been shown to have a potent phagocytic activity (42), we also investigated if rIFNa and rIFNγ had an effect on the capacity of IgM^+^IgD^+^ B cells to phagocytise microparticles. For this, PBLs were pre-treated with the rIFNs and after 72 h, the phagocytosis assay was conducted. Our results show that the pre-stimulation of IgM^+^IgD^+^ B cells with rIFNa significantly increased the percentage of phagocytic IgM^+^IgD^+^ B cells in the cultures when compared to either unstimulated cells or those treated with rIFNγ (Figure 4). Additionally, the MFI of internalized beads in blood IgM^+^IgD^+^ B cells was significantly higher in rIFNa-treated cells compared to controls (Figure 4), as was percentage of IgM^+^IgD^+^ B cells with a high number of ingested beads (highly phagocytic IgM^+^IgD^+^ B cells) (Figure 4). When we analyzed the effect of these cytokines on the IgM^−^IgD^−^ population, we found that in this case IFNa had positive effects on the percentage of phagocytic cells, whereas both cytokines increased the percentage of highly phagocytic cells in the cultures when compared to unstimulated cells.

### Type I and Type II IFN Increase IgM Secretion

**Figure 5:**
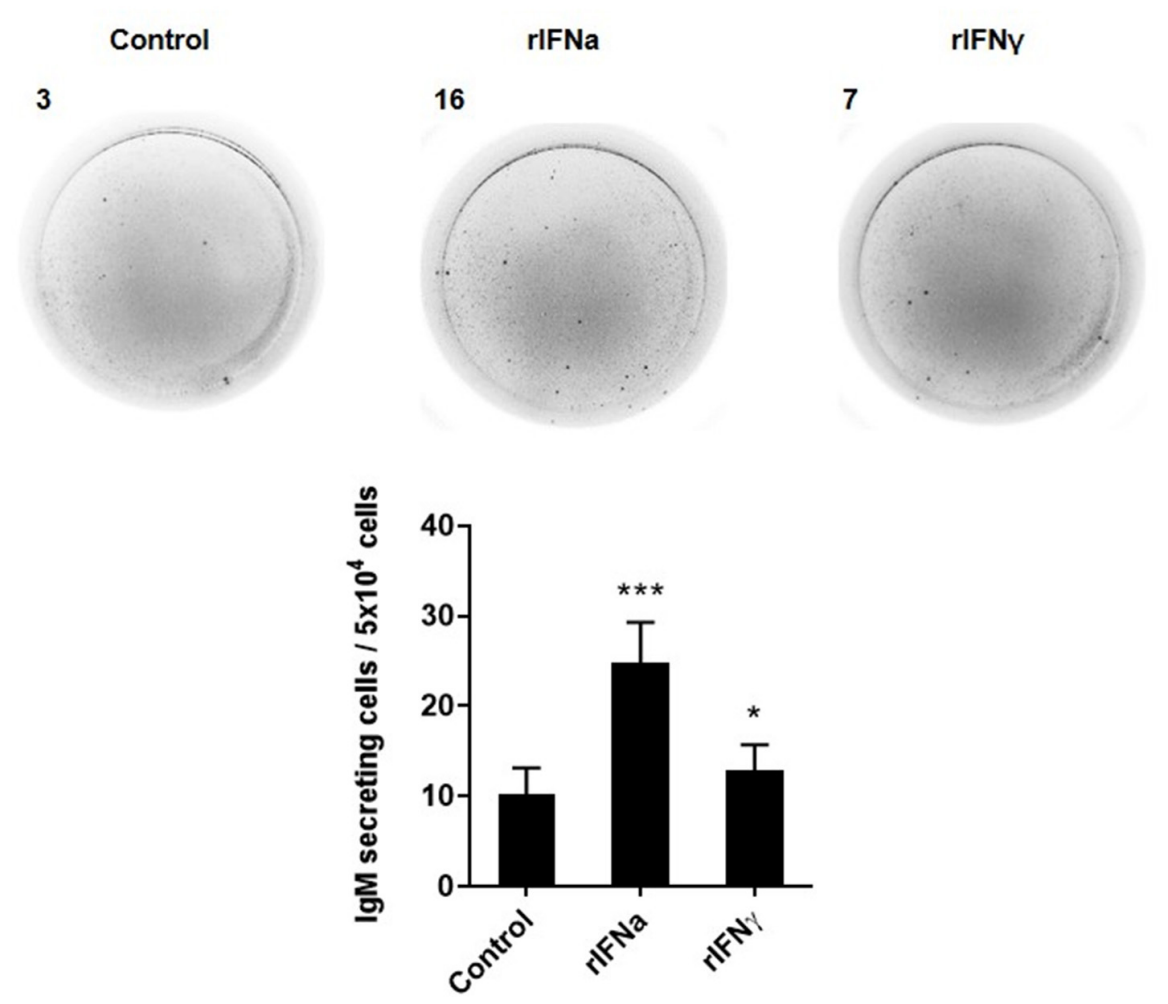
Effect of type I and type II IFNs on the differentiation of IgM^+^IgD^+^ B cells to IgM-secreting cells. PBLs were treated with 50 ng/ml rIFNa, 20 ng/ml rIFNγ or media alone (control) and incubated for 72 h at 20°C. Thereafter, cells were added to ELISPOT plates, previously coated with anti-IgM mAb (2 μg/ml), and incubated for a further 24 h. The number of IgM-secreting cells was then detected as described in Materials and Methods. Representative wells from one individual are shown along with the quantification of spot-forming cells (mean + SEM; *n* = 12). Asterisks denote significant differences between samples treated with rIFNs and control samples (*P ≤ 0.05, ***P ≤ 0.001).

We next studied, through ELISPOT, whether the rIFNs affected the capacity of rainbow trout blood B cells to secrete IgM, by determining the number of IgM-secreting cells (plasmablasts or plasma cells) in these PBL cultures. In this case, both rIFNa and rIFNγ significantly increased the number of IgM-secreting cells in the cultures after 3 days when compared to the number of IgM-secreting cells found in non-stimulated cultures (Figure 5), although the response to rIFNa was significantly higher than that of rIFNγ.

### rIFNs Have No Effect on the Antigen Presenting Capacities of Blood IgM^+^IgD^+^ B Cells

**Figure 6:**
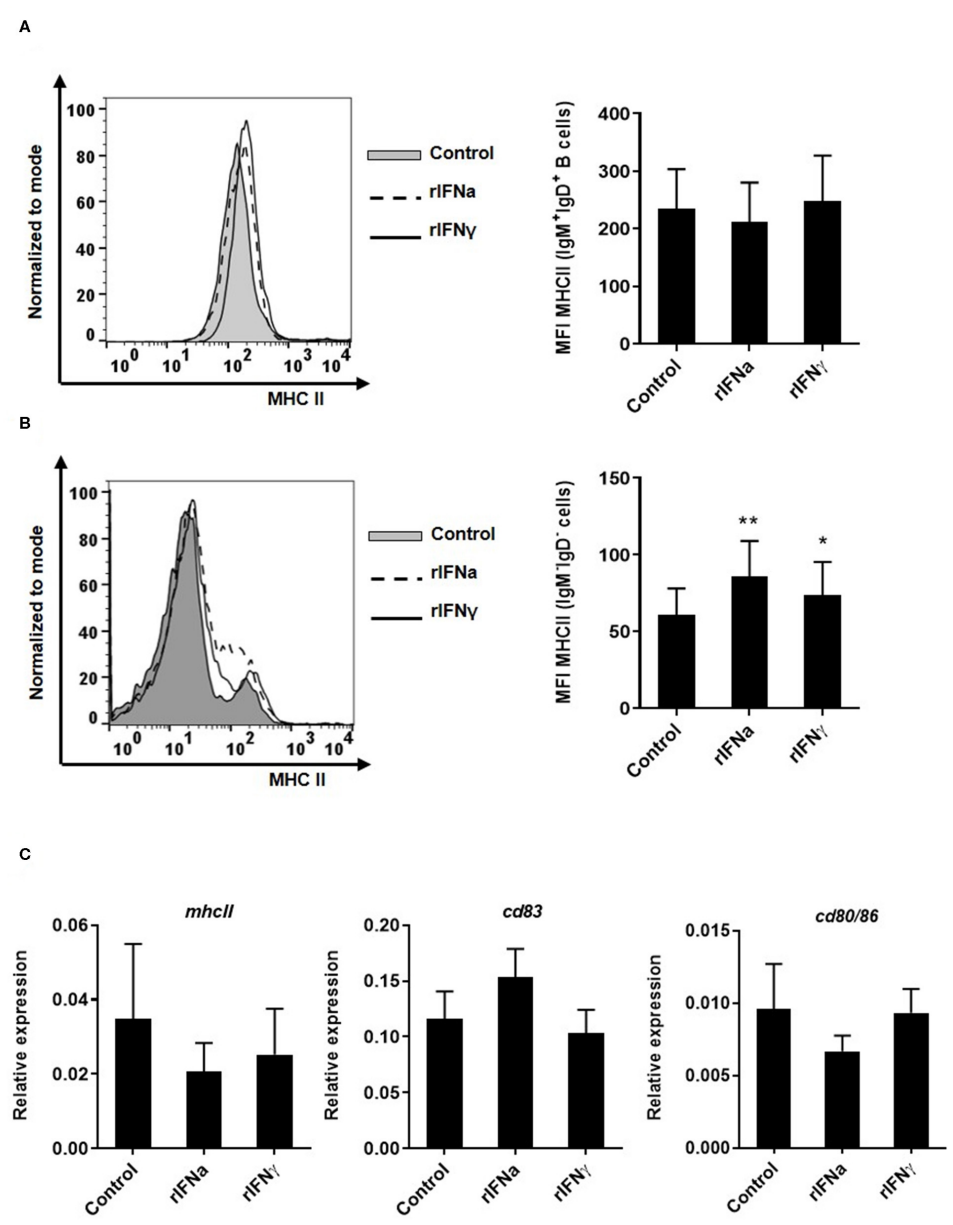
Effect of type I and type II IFNs on the antigen-presenting capacities of blood cells. PBLs were incubated with 50 ng/ml rIFNa, 20 ng/ml rIFNγ or media alone (control) for 72 h at 20°C. The level of MHC-II surface expression on IgM^+^IgD^+^ B cells and IgM^−^IgD^−^ cells was then measured via flow cytometry using a specific mAb against trout MHC-II. A representative histogram is shown together with a graph of the mean MHC-II MFI values for IgM^+^IgD^+^ B cells **(A)** or IgM^−^IgD^−^ cells **(B)** (mean + SEM; *n* = 9). In another experiment, blood leukocytes were treated with the rIFNs as described above and after 24 h IgM^+^IgD^+^ B cells were sorted by flow cytometry. RNA was extracted to determine the levels of transcription of *mhcII, cd83*, and *cd80/86* **(C)** by real time PCR. Gene expression data were normalized against the endogenous control *b-actin* and are shown as relative expression (mean + SEM; *n* = 10). Asterisks denote significant differences between samples treated with rIFNs and control samples (*P ≤ 0.05 and **P ≤ 0.01).

In mammals, IFNγ is one of the main cytokines involved in stimulating MHC class II expression in antigen presenting cells (56). Likewise, stimulation of rainbow trout RTS11 macrophages with 10 ng/ml of rIFNγ significantly increased MHC class II transcription between 24 and 72 h post-stimulation (51). In this context, we examined whether rIFNγ and rIFNa could modulate MHC II expression in blood IgM^+^IgD^+^ B cells, as they are also antigen presenting cells. We first evaluated MHC-II surface expression by flow cytometry using a specific anti-trout MHC-II. No significant modulation of MHC II surface expression (Figure 6A) or MHC II transcription (Figure 6C) was observed in IgM^+^IgD^+^ B cells. Additionally, we evaluated the effect of rIFNs on the level of transcription of CD80/86 and CD83, co-stimulatory molecules involved in antigen presentation, and again no effect was found (Figure 6C). Interestingly, both rIFNa and rIFNγ significantly increased the levels of surface MHC II in IgM^−^ cells in these cultures (Figure 6B). These results suggest that rIFNa and rIFNγ do not modulate the antigen presenting capacities of rainbow trout B cells, despite having effects on other antigen presenting cells, possibly monocytes/macrophages.

### Type I IFNs Induce the Transcription of Several Mx Proteins in Blood IgM^+^IgD^+^ B Cells

**Figure 7:**
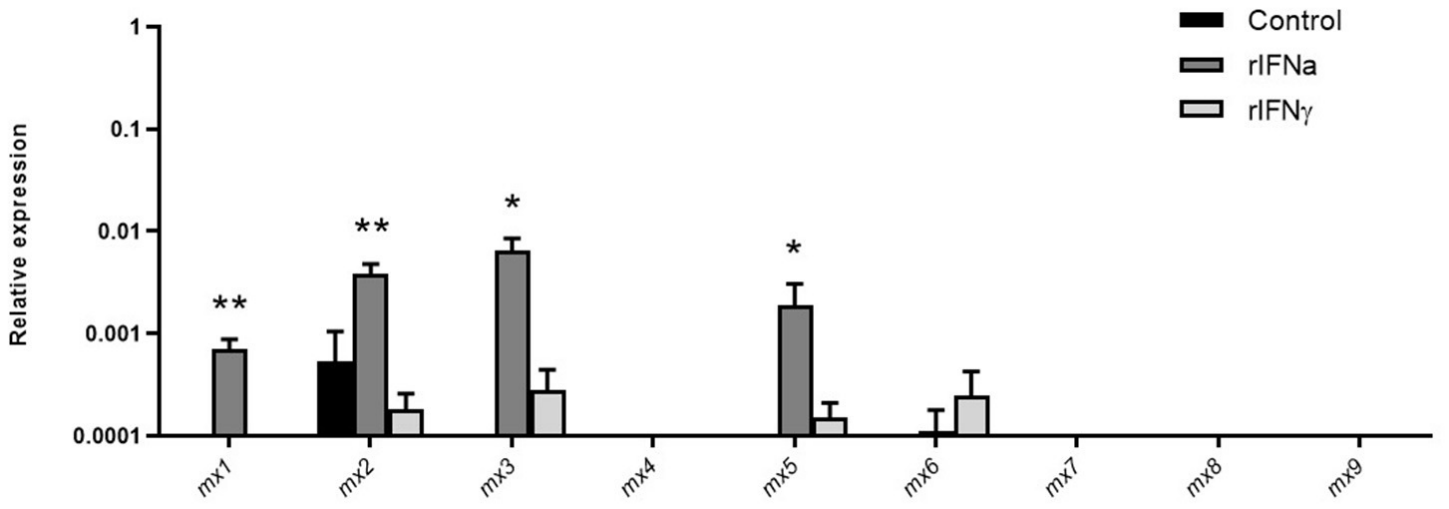
Induction of *mx* transcription in blood IgM^+^IgD^+^ B cells in response to type I or type II IFNs. Blood leukocytes were incubated with 50 ng/ml rIFNa, 20 ng/ml rIFNγ or media alone (control) at 20°C. After 24 h, IgM^+^IgD^+^ B cells were sorted by flow cytometry and RNA extracted to determine the levels of transcription of the different *mx* genes present in rainbow trout (*mx1*-*mx9*). Gene expression data were normalized against the endogenous control *b-actin* and are shown as relative expression (mean + SEM; *n* = 10). Asterisks denote significant differences between samples treated with rIFNs and control samples (*P ≤ 0.05 and **P ≤ 0.01).

Mx proteins are dynamin-like GTPases, which play an important role in antiviral immunity (57) Mammalian Mx expression is generally induced by type I and type III IFNs but not by type II IFN (58), although some exceptions to this general rule have been found recently in teleost fish (59). Interestingly, it has also been shown recently that the Mx family of proteins has expanded in salmonids, with 10 different *mx* genes present in Atlantic salmon and 9 in rainbow trout (48). However, to date, Mx expression has not been studied to our knowledge in fish B cells. Our results demonstrate that rIFNa significantly induced the transcription of Mx1, Mx3, and Mx5 (Figure 7) in sorted blood IgM^+^IgD^+^ B cells and significantly up-regulated the mRNA levels of Mx2, constitutively transcribed by these cells (Figure 7). rIFNγ, on the other hand, had no significant effects on Mx transcription (Figure 7).

## Discussion

In addition to the well-known direct antiviral effects of type I IFNs and the ability of type II IFN to modulate antigen presentation, it has now become evident that IFNs are also involved in the regulation of lymphocyte development and differentiation. However, it is generally believed that B cells are mainly modulated by type I IFN, while T cells are mostly responsive to type II IFN (60). To investigate whether fish B cells are also responsive to type I IFNs, we have studied the effects of a model type I trout IFN protein (IFNa) on naïve blood B cells and compared the effects to those elicited by type II IFN produced under the same conditions. In these assays, the optimal concentrations for each rIFN were previously established and were different for each protein (50 ng/ml for IFNa and 20 ng/ml for IFNγ), in concordance with previous publications (47, 48, 51). Having said this, all the assays described in the current study were initially performed with IFNγ at 50 ng/ml but no significant differences with the results presented here were found (Figure S2).

The first effect that was clearly visible when PBLs were incubated with IFNa was that both the percentage of IgM^+^IgD^+^ B cells and the absolute number of IgM^+^IgD^+^ B cells increased significantly in the cultures in the presence of the cytokine after 72 h. As many other leukocyte types were also present in these cultures, it could have been possible that this increase in B cells was a consequence of IFNa affecting other cell types that afterwards activated B cells. For example, mammalian type I IFN, produced during the course of a viral infection, can activate DCs and macrophages to produce BAFF and APRIL which in turn activate B cells (19). Hence, to rule out possible indirect effects in our experiments, we sorted trout blood B cells and then stimulated them with rIFNa. In this case, similar positive effects on B cell survival were observed, pointing to a direct effect of the cytokine. This increase in the number of B cells in PBL cultures was not consequence of IFNa-induced lymphoproliferative effects as established through *in vitro* proliferation assays. In mammals, type I IFN rescues B cells from Fas induced spontaneous apoptosis through a mechanism involving phosphorylation of AKT and up-regulation of pro-survival molecules (14, 15). Hence, we investigated whether this could be the origin of the increased B cell survival in blood leukocyte cultures. Our results demonstrated that IFNa provoked a significant down-regulation of B cell spontaneous apoptosis, suggesting a similar mechanism to that reported in mammals. rIFNγ, on the other hand, only increased slightly the absolute number of IgM^+^IgD^+^ B cells in the cultures, but had no effect on the percentage of IgM^+^IgD^+^ B cells. Similarly, there was no effect of rIFNγ when cells were sorted and then incubated with the cytokine, nor on B cell apoptosis. Therefore, rIFNγ may be exerting some minor effects on B cell viability through indirect mechanisms, but at levels much lower than those described here for rIFNa. In contrast, rIFNγ had significant effects on the phagocytic capacity and the levels of surface MHC II expression of IgM^−^IgD^−^ leukocytes present in the cultures.

In mammals, type I IFN acts as an amplifier of BCR signaling during viral infections, by taking the B cells to a state of partial activation in which cells have a higher sensitivity to further stimulation through the BCR (14). Thus, type I IFN significantly enhances responses to BCR ligation that include proliferation, calcium flux, IgM internalization and induction of activation markers (14). In rainbow trout, however, rIFNa did not synergize with BCR signaling to induce proliferation. The reason for this different response of trout B cells could be linked to the fact that fish B cells do not proliferate in response to BCR ligation alone, unlike mammalian conventional B2 cells but similar to mammalian B1 cells (43). In fact, there are many other functional and phenotypical similarities between fish B cells and mammalian innate B1 cells that include a strong phagocytic capacity, longer survival in cell culture, low IgD surface expression or expression of B1-specific markers (43). Interestingly, trout rIFNa was shown to up-regulate one of these innate features of fish B cells, their phagocytic activity, significantly increasing the number of phagocytic B cells in the cultures and the percentage of B cells with a higher number of phagocytized beads. In this context, it would be interesting to investigate in mammals the effects of type I IFN in mammalian B1 cells, and determine for example, whether type I IFN also induces the phagocytic capacity of these cells as established for fish B cells.

Mammalian type I IFN has been repeatedly reported to increase antibody responses *in vivo* (61, 62). In these experiments, the increased antibody titers were dependent on B cells being able to directly respond to IFNα/β (61, 62), demonstrating a direct effect of type I IFN on B cell differentiation. To investigate if rIFNs on their own were capable of inducing the differentiation of fish naïve B cells, we determined the number of IgM-secreting cells in blood leukocyte cultures after 72 h of exposure. In this case, both rIFNa and rIFNγ significantly increased the number of plasmablasts/plasma cells in the cultures, although as observed throughout all the experiments, the effects of type I IFN were significantly higher than those of type II IFN. It might be possible that this increase in the number of IgM-secreting cells is a consequence of a general increased survival of B cells in the cultures, however, in agreement with our results, Atlantic salmon IFNa has been shown to be a potent molecular adjuvant for a DNA vaccine against infectious salmon anemia virus (ISAV), significantly increasing specific IgM titers to the vaccine (63). Furthermore, when a transcriptomic analysis of the local response to this plasmid coding for IFNa was undertaken in Atlantic salmon, IgM, IgD, and IgT were among the genes that were strongly up-regulated (64).

Mx genes are present in almost all vertebrates, usually as one to three copies. In mammals, these multiple copies are generally closely related and have arisen from local gene duplications (65). However, possibly as a consequence of the third teleost-specific WGD and a later salmonid-specific WGD, the Mx gene family, as with many other gene families, has undergone extensive diversification, with 6–10 different genes identified in salmonids (48, 59). In rainbow trout, the 9 Mx genes present were constitutively transcribed by RTS11 macrophages, and among them, Mx1-7 were strongly induced by IFNa while only Mx5 and Mx6 increased their mRNA levels in response to IFNγ (48). Similarly, in Atlantic salmon, *mx* genes showed a differential responsiveness to type I and II IFNs, with those on Chr12 being highly induced by type I IFNs and those on Chr25 being more strongly induced by IFNγ than by type I IFN (48, 59). However, to our knowledge, which *mx* genes are expressed in B cells had never been addressed in teleost fish. Our results suggest that Mx4, Mx7, Mx8, and Mx9 are not produced by blood B cells, while Mx1, Mx2, Mx3, and Mx5 were strongly up-regulated in response to rIFNa (but not IFNγ). Whether this induction of Mx expression within B cells limits the capacity of viruses to replicate within these cells is something that should be investigated further, given that a direct antiviral activity has not yet been demonstrated for trout Mx proteins.

In conclusion, we report a previously undescribed role for teleost type I IFN in the regulation of B cell responses. Our results demonstrate that, as occurs in mammals, IFNa increased the survival of naïve B cells by rescuing them from spontaneous apoptosis and increased IgM secretion. However, unlike mammalian B2 cells, fish B cells treated with type I IFN did not have an increased responsiveness to BCR stimulation. A possible explanation for this different response might be linked to the fact that fish B cells react differently to BCR stimulation than mammalian conventional B cells. Furthermore, given that fish B cells actually retain some innate functions such as a strong phagocytic capacity, we have also established that type I IFN regulates this activity in fish B cells. Finally, the fact that several *mx* genes are induced within B cells as a response to rIFNa points to an effect limiting the capacity of viruses to replicate within B cells. Our results provide further evidence of the interplay between cytokines of the innate immune system and cells of the adaptive immune system, which is an essential factor in the regulation of the immune response.

## Data Availability Statement

The datasets generated for this study are available on request to the corresponding author.

## Ethics Statement

The animal study was reviewed and approved by INIA Ethics Committee.

## Author Contributions

TW produced the recombinant IFNs. OB performed and analyzed all the experiments with help from IS and PD-R. EM provided support with all flow cytometry experiments and performed the cell sortings. CT conceived the work and designed the experiments with help from TW, CS, OB, and PD-R and wrote the main body of the paper with contributions from all other authors. All authors contributed to the article and approved the submitted version.

## Conflict of Interest

The authors declare that the research was conducted in the absence of any commercial or financial relationships that could be construed as a potential conflict of interest.
