# Corrigendum: Type I Interferon Regulates the Survival and Functionality of B Cells in Rainbow Trout

**DOI:** 10.3389/fimmu.2021.778085

**Published:** 2021-10-18

**Authors:** Ottavia Benedicenti, Tiehui Wang, Esther Morel, Christopher J. Secombes, Irene Soleto, Patricia Díaz-Rosales, Carolina Tafalla

**Affiliations:** ^1^ Animal Health Research Center (CISA-INIA), Madrid, Spain; ^2^ Scottish Fish Immunology Research Centre, School of Biological Sciences, University of Aberdeen, Aberdeen, United Kingdom

**Keywords:** teleost fish, B cells, interferon (IFN), IgM, phagocytosis

In the original article, there was a mistake in **Figure 1** as published. Although the numbers were correct, all dot plots in **Figure 1A** were the same. The corrected **Figure 1** appears below.

**Figure 1:**
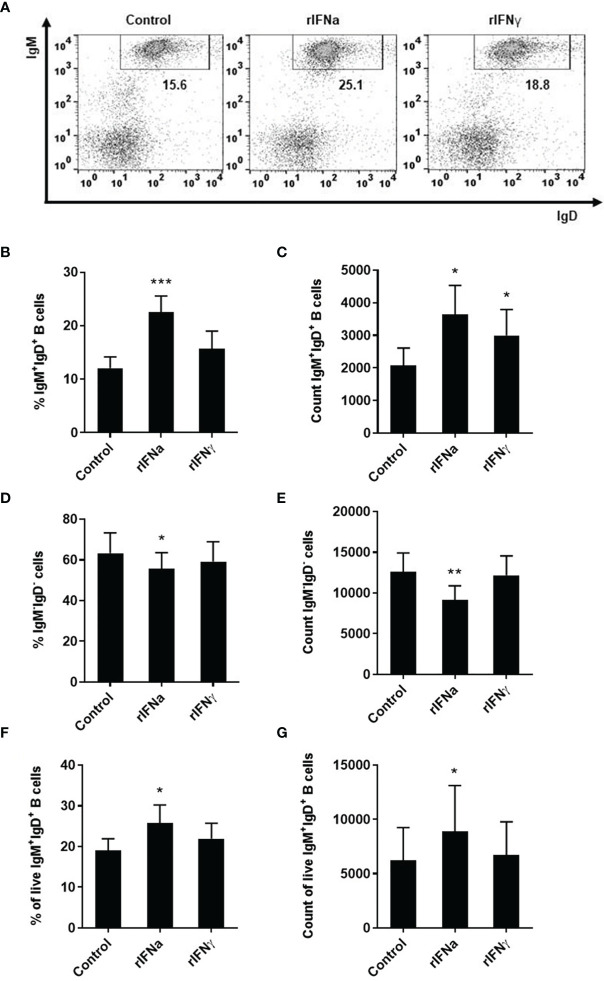
Survival of blood IgM+IgD+ B cells in response to type I and type II IFNs. PBLs were stimulated with 50 ng/ml rIFNa, 20 ng/ml rIFNg or media alone (control) and cultured at 20°C for 72 h. Leukocytes were then labeled with specific monoclonal antibodies against trout IgM and IgD and analyzed by flow cytometry. Cells were gated on the basis of their FSC and SSC and percentages of IgM+IgD+ cells determined on singlet and live (DAPI negative) cells. Representative dot plots from one individual fish are shown **(A)** along with mean percentages and total number of cells detected for IgM+IgD+ B cells **(B)** and IgM−IgD− cells **(C)** (mean + SEM; n = 9). In an independent experiment, B cells were sorted from blood leukocytes using a biotinyilated Fab fragment of anti-IgM 1.14 and then incubated with the rIFNs as described above. After 72 h, the percentage of live IgM+IgD+ B cells and the total number of live IgM+IgD+ B cells determined by flow cytometry as described in the Materials and Methods section (mean + SEM; n = 7) **(D)**. Asterisks denote significant differences between samples treated with rIFNs and control samples (*P ≤ 0.05, **P ≤ 0.01, ***P ≤ 0.001).

The authors apologize for this error and state that this does not change the scientific conclusions of the article in any way. The original article has been updated.

## Publisher’s Note

All claims expressed in this article are solely those of the authors and do not necessarily represent those of their affiliated organizations, or those of the publisher, the editors and the reviewers. Any product that may be evaluated in this article, or claim that may be made by its manufacturer, is not guaranteed or endorsed by the publisher.

