# Supplementary material for: Type I Interferon Regulates the Survival and Functionality of B Cells in Rainbow Trout
Source: Front Immunol. 2020 Jul 9;11:1494. doi: 10.3389/fimmu.2020.01494 (PMC7363951; doi:10.3389/fimmu.2020.01494)
Supplement: Supplementary file 1 [file Presentation_1.PPTX]

## Slide 1
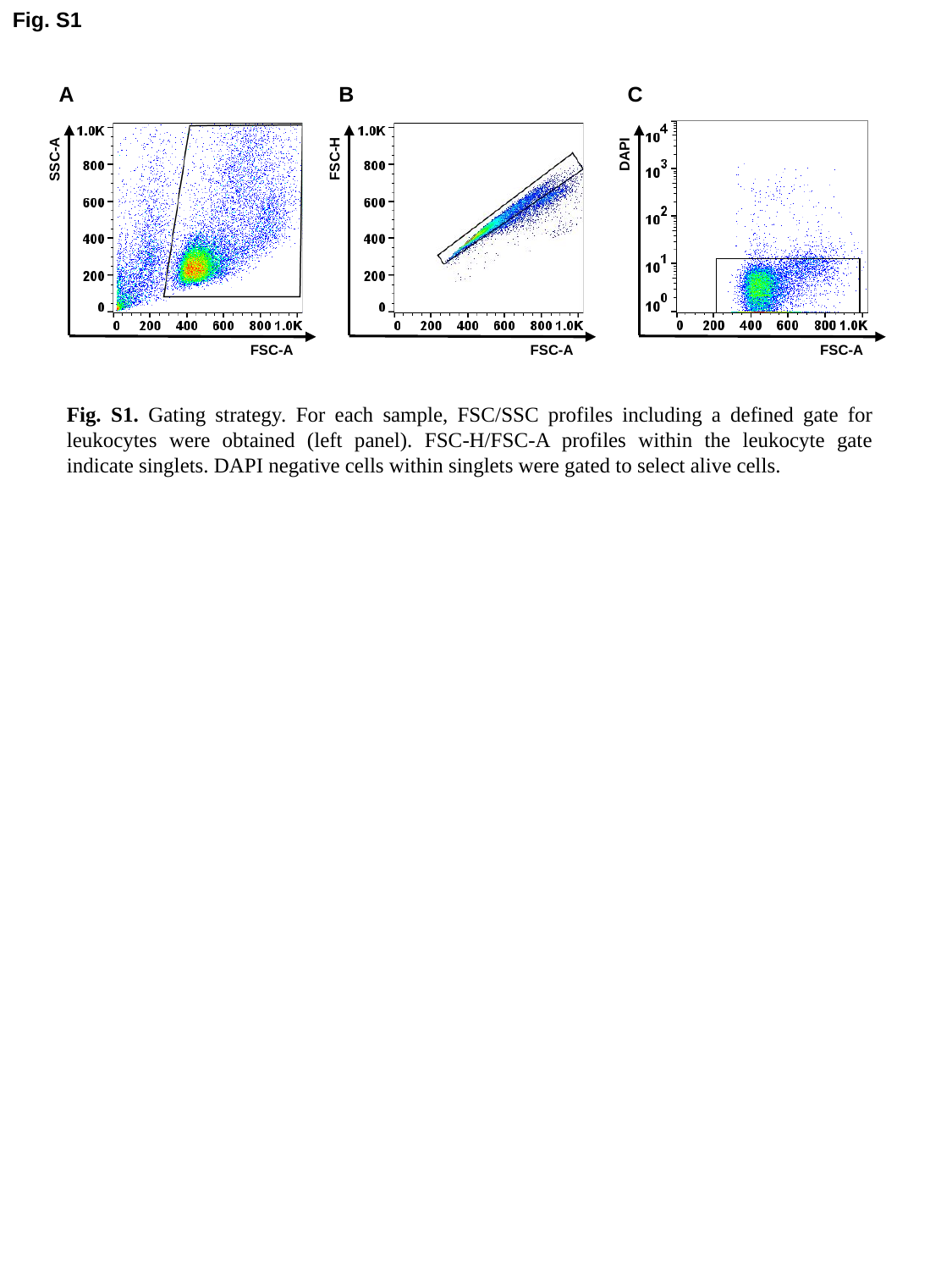

Fig. S1
A
B
C
SSC-A
FSC-H
DAPI
FSC-A
FSC-A
FSC-A
Fig. S1. Gating strategy. For each sample, FSC/SSC profiles including a defined gate for leukocytes were obtained (left panel). FSC-H/FSC-A profiles within the leukocyte gate indicate singlets. DAPI negative cells within singlets were gated to select alive cells.

## Slide 2
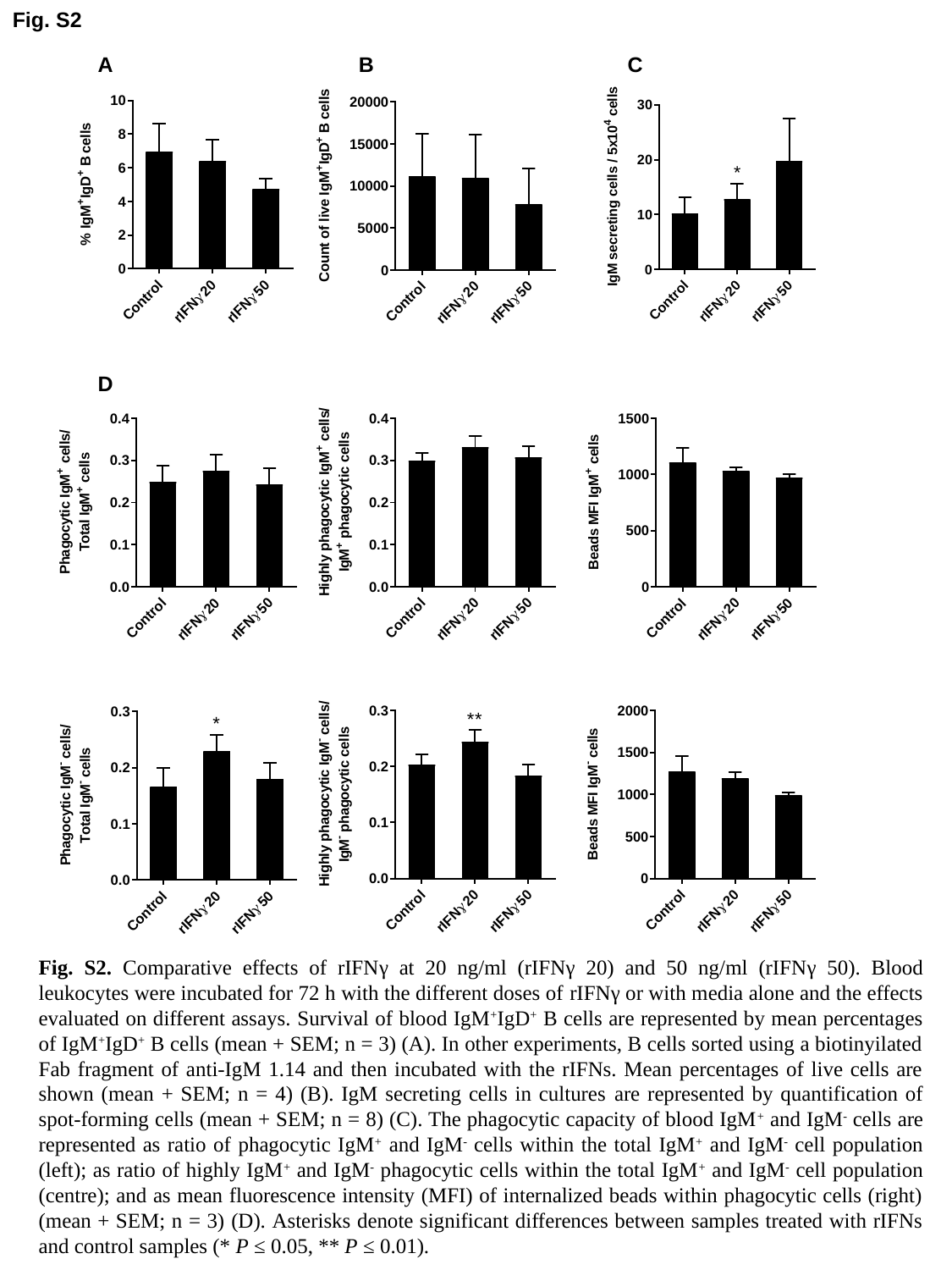

Fig. S2
A
B
C
D
Fig. S2. Comparative effects of rIFNγ at 20 ng/ml (rIFNγ 20) and 50 ng/ml (rIFNγ 50). Blood leukocytes were incubated for 72 h with the different doses of rIFNγ or with media alone and the effects evaluated on different assays. Survival of blood IgM+IgD+ B cells are represented by mean percentages of IgM+IgD+ B cells (mean + SEM; n = 3) (A). In other experiments, B cells sorted using a biotinyilated Fab fragment of anti-IgM 1.14 and then incubated with the rIFNs. Mean percentages of live cells are shown (mean + SEM; n = 4) (B). IgM secreting cells in cultures are represented by quantification of spot-forming cells (mean + SEM; n = 8) (C). The phagocytic capacity of blood IgM+ and IgM- cells are represented as ratio of phagocytic IgM+ and IgM- cells within the total IgM+ and IgM- cell population (left); as ratio of highly IgM+ and IgM- phagocytic cells within the total IgM+ and IgM- cell population (centre); and as mean fluorescence intensity (MFI) of internalized beads within phagocytic cells (right) (mean + SEM; n = 3) (D). Asterisks denote significant differences between samples treated with rIFNs and control samples (* P ≤ 0.05, ** P ≤ 0.01).

## Slide 3
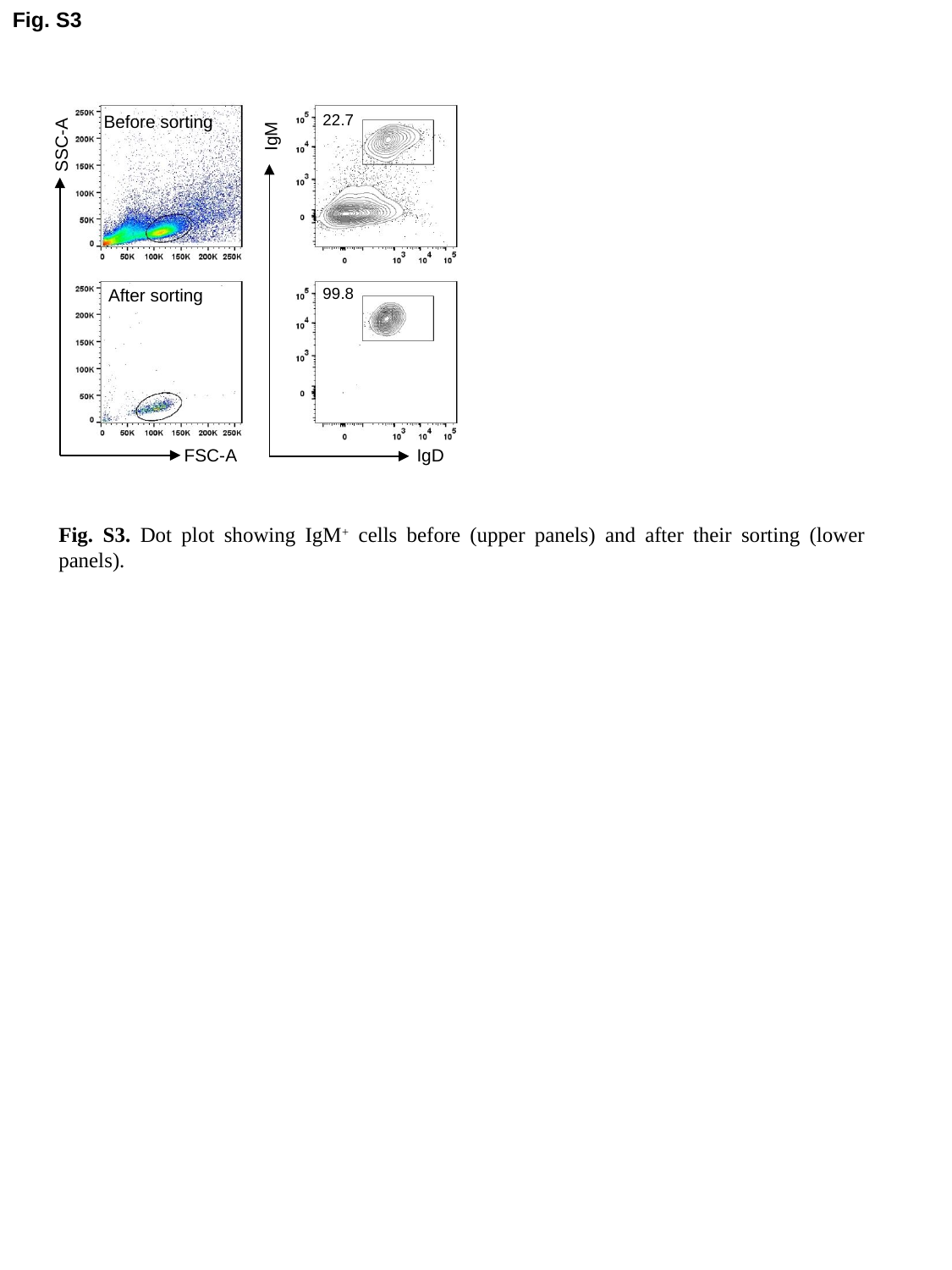

Fig. S3
IgM
IgD
SSC-A
FSC-A
Before sorting
22.7
99.8
After sorting
Fig. S3. Dot plot showing IgM+ cells before (upper panels) and after their sorting (lower panels).

## Slide 4
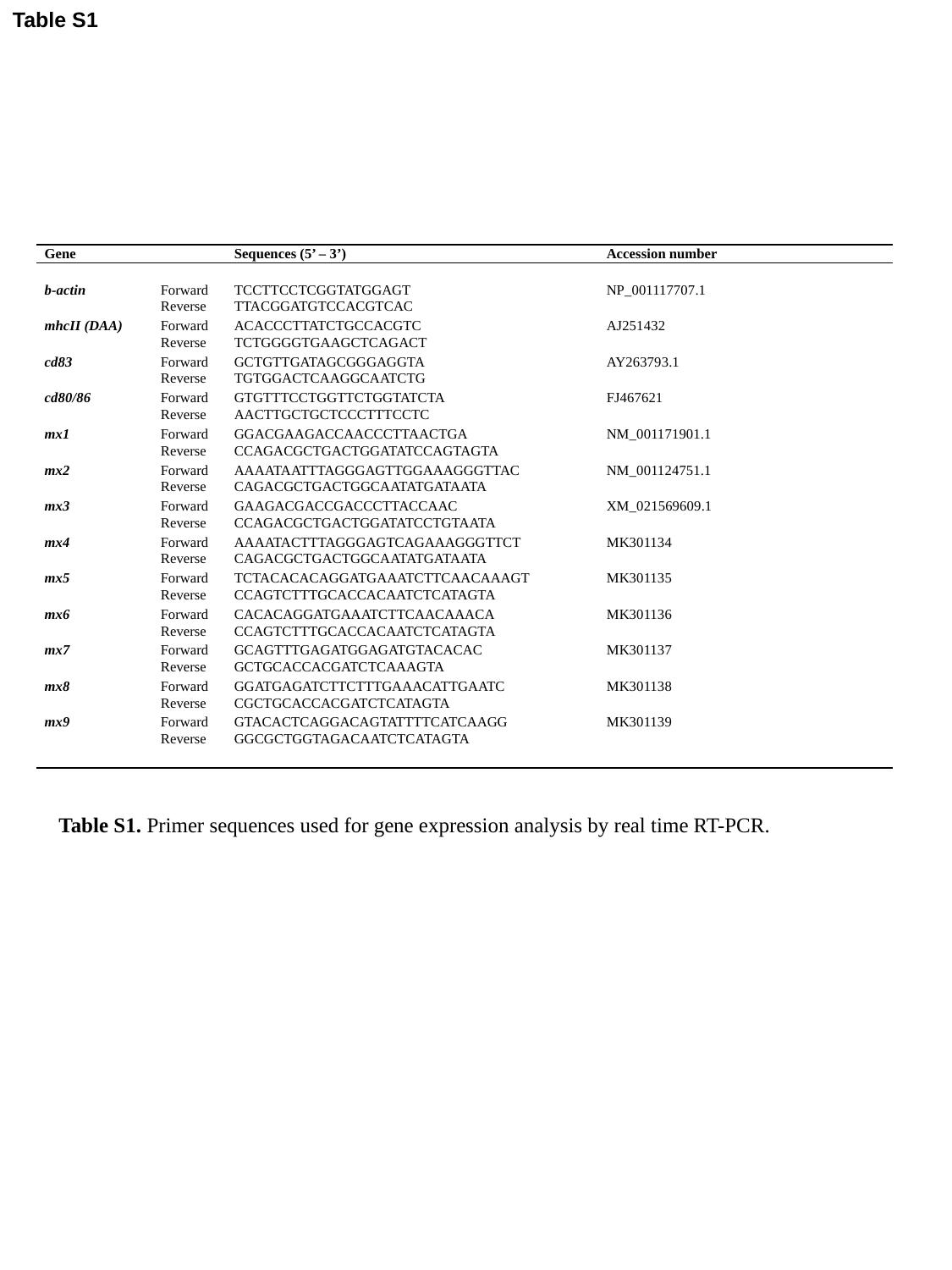

Table S1
| Gene | | Sequences (5’ – 3’) | Accession number |
| --- | --- | --- | --- |
| | | | |
| b-actin | Forward Reverse | TCCTTCCTCGGTATGGAGT TTACGGATGTCCACGTCAC | NP\_001117707.1 |
| mhcII (DAA) | Forward Reverse | ACACCCTTATCTGCCACGTC TCTGGGGTGAAGCTCAGACT | AJ251432 |
| cd83 | Forward Reverse | GCTGTTGATAGCGGGAGGTA TGTGGACTCAAGGCAATCTG | AY263793.1 |
| cd80/86 | Forward Reverse | GTGTTTCCTGGTTCTGGTATCTA AACTTGCTGCTCCCTTTCCTC | FJ467621 |
| mx1 | Forward Reverse | GGACGAAGACCAACCCTTAACTGA CCAGACGCTGACTGGATATCCAGTAGTA | NM\_001171901.1 |
| mx2 | Forward Reverse | AAAATAATTTAGGGAGTTGGAAAGGGTTAC CAGACGCTGACTGGCAATATGATAATA | NM\_001124751.1 |
| mx3 | Forward Reverse | GAAGACGACCGACCCTTACCAAC CCAGACGCTGACTGGATATCCTGTAATA | XM\_021569609.1 |
| mx4 | Forward Reverse | AAAATACTTTAGGGAGTCAGAAAGGGTTCT CAGACGCTGACTGGCAATATGATAATA | MK301134 |
| mx5 | Forward Reverse | TCTACACACAGGATGAAATCTTCAACAAAGT CCAGTCTTTGCACCACAATCTCATAGTA | MK301135 |
| mx6 | Forward Reverse | CACACAGGATGAAATCTTCAACAAACA CCAGTCTTTGCACCACAATCTCATAGTA | MK301136 |
| mx7 | Forward Reverse | GCAGTTTGAGATGGAGATGTACACAC GCTGCACCACGATCTCAAAGTA | MK301137 |
| mx8 | Forward Reverse | GGATGAGATCTTCTTTGAAACATTGAATC CGCTGCACCACGATCTCATAGTA | MK301138 |
| mx9 | Forward Reverse | GTACACTCAGGACAGTATTTTCATCAAGG GGCGCTGGTAGACAATCTCATAGTA | MK301139 |
| | | | |
Table S1. Primer sequences used for gene expression analysis by real time RT-PCR.
